# Performance of Self-Healing Cementitious Composites Using Aligned Tubular Healing Fiber

**DOI:** 10.3390/ma14206162

**Published:** 2021-10-18

**Authors:** Ru Mu, Dogniman Landry Soro, Xiaowei Wang, Longbang Qing, Guorui Cao, Shaolin Mei, Yongshuai Liu

**Affiliations:** School of Civil and Transportation Engineering, Hebei University of Technology, Tianjin 300401, China; ru_mu@hotmail.com (R.M.); slandry228@gmail.com (D.L.S.); qing@hebut.edu.cn (L.Q.); 201531603006@stu.hebut.edu.cn (G.C.); msl1997_svip@163.com (S.M.); 202021601038@stu.hebut.edu.cn (Y.L.)

**Keywords:** aligned healing fibers, randomly distributed healing fibers, aligned hybrid fibers with double steel fibers, aligned hybrid fibers with single steel fiber

## Abstract

From the perspective of improving the self-healing method in construction, a tubular healing fiber was adopted as a container to improve the encapsulation capacity, which was available using a micro-capsule as a container. Knowing the direction of the stresses to which structure members are subjected, this research investigated the influence of aligning tubular healing fibers parallel to intended stress into a cementitious composite to increase the self-healing capability. For that, a healing agent was encapsulated into a tubular healing fiber made with polyvinylidene of fluoride resin (PVDF). Then, the healing fiber was combined with steel fibers to align both fibers together parallel to the direction of an intended splitting tensile stress when subjected to a magnetic field in a cylindrical cementitious composite. The alignment method and the key point through which the alignment of the healing fibers could efficiently improve autonomic self-healing were investigated. Since the magnetic field is known to be able to drag steel to an expected direction, steel fibers were combined with the healing fibers to form a hybrid fiber that aligned both fibers together. The required mixture workability was investigated to avoid the sinking of the healing fibers into the mixture. The healing efficiency, according to the orientation of the healing fibers in the composite matrix, was evaluated through a permeability test and a repetitive splitting tensile test. The aligned healing fibers performed better than the randomly distributed healing fibers. However, according to the healing efficiency with aligned healing fibers, it was deduced that the observed decreasing effect of the container’s alignment on the specimen’s mechanical properties was low enough to be neglected.

## 1. Introduction

Concrete is one of the most widely used materials for construction, mainly because of its excellent ability to withstand stresses and the possibility of manufacturing it according to the intended properties. This was proved through the evaluation of the world’s annual production of concrete in 2015 to be more than 10 billion tons, along with the prediction that it is expected to reach 18 billion tons by 2050 [1]. However, concrete is known to be less resilient toward tensile stresses, which makes it vulnerable to cracks [2]. To solve crack problems in cementitious materials, regular maintenance of structures is usually required, which creates huge costs for the owners [3]. Through a study, it was indicated that in 2020, the annual maintenance cost worldwide was estimated to be $147 million when the US alone spent approximately $21 billion for maintenance [3]. Therefore, to efficiently overcome the crack problems in cementitious materials and reduce the structures’ maintenance costs, the development of crucial methods, such as self-healing in cementitious materials has been implemented [4]. 

The self-healing in construction means allowing structures to activate at a moment a mechanism (natural or human-made) to prevent micro-crack development [5]. The self-healing method can be mainly achieved in two ways, which are the autogenic and autonomic processes [6]. The autogenic method is mainly based on regular natural hydrations of non-hydrated cement nuclei remaining in the cementitious matrix, while the autonomic method is based on chemical or biological processes [7]. Additionally, it has also been indicated that the success of the autonomic method requires specific criteria, which include: a sufficient encapsulating volume of the healing agent, high hardening time of the healing agent, good adhesion between the container’s shell with the cementitious matrix, good resistance of the container’s shell against chemical attacks, and good mechanical performances [7]. According to the literature, the autonomic self-healing method by encapsulation has been indicated as the most efficient, since it allows users to recover as much as possible of the initial mechanical and durability properties of the material composite [3]. To realize an autonomic healing method, microcapsules were the first means used as a container for the encapsulation of the external component (healing agent or bacteria). However, to improve the encapsulation capacity, containers with tubular shapes were then adopted. Thus, to fill the requirements of the autonomic method, tubular containers with different base materials have been investigated, such as tubular fibers made with polyvinylidene of fluoride resin (PVDF) [7], glass fiber [8], polypropylene tube [9], polyurethane tube [9], and so on. Crack width has been indicated as a significant factor that influences the healing effectiveness, in addition to the volume capacity of the container used for self-healing [10]. It has also been stated that the container’s content ratio in composite material and the orientation angle in the case of tubular containers can contribute to increasing the healing capacity [10]. A preventive point has indicated that although the container’s content ratio in a material can increase the effectiveness of crack healing, this content can also decrease the mechanical properties of that material [11]. However, it is emphasized that the influence of the tubular fiber’s content on the material’s mechanical properties can be neglected compared to the healing efficiency [11]. In addition to the decrease in the material’s mechanical properties caused by the tubular fiber’s content ratio, it may not be enough to efficiently improve the healing capability if, despite the encapsulation capacity and the container’s content ratio, it is not ensured to have enough containers across the cracks section. Therefore, to efficiently improve the healing capability without necessarily using a high container content, which could affect the material’s mechanical properties, aligning tubular containers parallel to the direction of the intended stress into the structure can eventually ensure the crack crosses several containers, increasing the number of containers across the crack-section and, at the same time increasing the available volume of healing agent. 

So, it is in this manner our research inscribes itself, planning to align a healing fiber parallel to the direction of an intended splitting tensile stress, using a magnetic field in a cementitious composite for autonomic self-healing. A container with a tubular shape was used to ensure a good enough encapsulation capacity, made with polyvinylidene of fluoride resin as the basis material, especially because of the proven properties this material provides. These properties include good adhesion, good resistance to high temperatures and chemical attacks, as well as good mechanical properties, thus meeting the container’s requirements for effective autonomic self-healing. Additionally, polyurethane was used as the healing agent, due to the reliable properties it provides, such as good mechanical and durability properties, which can allow recovering as much as possible the initial properties of the material. A high hardening time contributes to ensuring good conservation of the healing agent into the composite material and a good flow after having been released.

## 2. Materials and Mixtures

**Figure 1:**
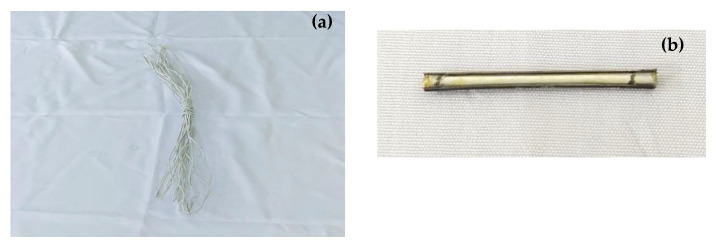
Containers preparation: (**a**) healing fiber, (**b**) hybrid fiber.

An ordinary Portland cement P∙O 42.5, natural river sand with 2.6 as fineness modulus, and polycarboxylate (PC) as a superplasticizer were used for the manufacturing of all the specimens in this work. The mixture used to make the specimens was cement: sand: water = 651:1302:235 kg/m^3^. The water to cement ratio was 0.36 (as shown in Table 1). A polyurethane glue (PU) manufactured by Shanghai Fine Stationery Co., Ltd. was used as the healing agent for the self-healing process due to its suitable properties. A tubular healing fiber supplied by the School of Material Science and Engineering, Tianjin Polytechnic University (Tianjin 300387#, China), made with polyvinylidene of fluoride resin (PVDF) using a dry–wet method, spinning as described in prior works [12], was the container used to encapsulate the healing agent, as shown in Figure 1a. The tubular healing fiber with 0.6 mm as inner diameter and a density of 1.78 g/cm^3^, has mainly been made according to a coagulation water bath temperature of 25 ± 1 °C with PVDF contents of 30%, the molecular weight of polymer 570–600 kDa [12]. Cylindrical steel fibers with 0.5 mm as diameter and 30 mm as length were manually combined with the healing fibers using an ordinary super glue supplied on the market, which formed a hybrid fiber as shown in Figure 1b, to enable both fibers (steel fiber and healing fiber) to be aligned together according to the intended direction of the applied electromagnetic field on the mixture. Additionally, the steel fibers were also used in the specimen matrix to be able to control the development of the crack width during the splitting tensile test.

**Figure 2:**
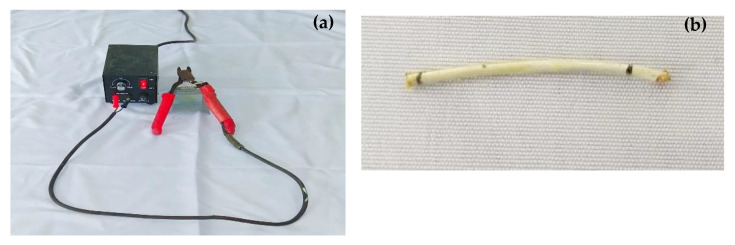
Encapsulation equipment: (**a**) hot scissors equipped with a battery, (**b**) sample of healing fiber.

To allow the cementitious composites to self-heal the induced cracks during the tests, the polyurethane glue was encapsulated into healing fibers before being integrated into the composite matrix. The encapsulation process of the healing agent was realized by using a certain length of healing fibers that were sealed at one side, then the healing agent was injected into the healing fibers using a syringe. The healing agent was prepared using the polyurethane glue with a diluent from Tianjin Zhonghe Shengtai Chemical Co., Ltd., (Tianjin, China) according to a ratio of 2:1, which allowed us to obtain a glue sufficiently fluid to be easily injected into the healing fibers. After that, a hot scissor equipped with a battery as shown in Figure 2a was used to manually cut and seal at the same time the healing fibers containing the healing agent within several fibers of 30 mm length each as shown in Figure 2b.

## 3. Alignment of Tubular PVDF Fibers Using Electromagnetic Field

### 3.1. Optimal Required Conditions

**Figure 3:**
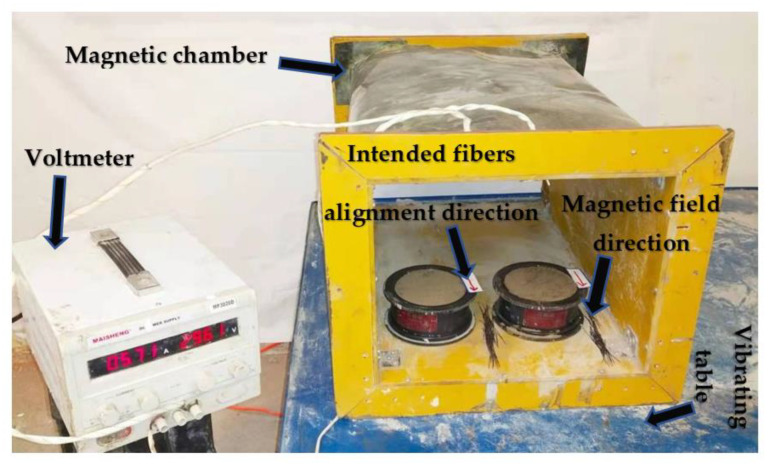
Equipment for the alignment process.

Based on the ability of a magnetic field to drag steel, this method has been thought of as a means to align the healing fibers into a cementitious mixture, combining them with steel fibers to form a hybrid fiber. The process of using a magnetic field to align steel fibers into a cementitious mixture was demonstrated in a prior study and indicated the necessary requirements for the alignment process [13]. Thus, it has been shown that during the alignment process of steel fiber into a cementitious mixture, only the magnetic field and the viscous force of the mixture act in the steel fiber’s orientation [13]. However, it has been emphasized that for an effective alignment process, the viscous force, which is related to the mixture workability, should be significantly low to enable the steel fibers to be dragged by the magnetic field [13]. In this way, in this research, before conducting the alignment process, the optimal requirements, such as the suitable workability of the mixture and the amount of steel fiber required to combine with each healing fiber, have been investigated. For that, the investigations on the alignment efficiency of hybrid fibers into a cementitious mixture were realized according to different mixtures’ workability and the amount of the combined steel fiber with each healing fiber. A magnetic chamber made up of a solenoid coil wound, mounted on a vibrating table and connected to a voltmeter, was the instrument adopted to achieve the automatic alignment process, as shown in Figure 3. Two main types of specimen were made, namely, specimens containing aligned hybrid fiber (combination of healing fiber with single steel fiber) in a cementitious composite (AHFCC-1SF) and specimens containing aligned hybrid fibers (combination of healing fiber with double steel fibers) in a cementitious composite (AHFCC-2SF).

In order to realize the alignment process of the hybrid fibers, both types of the specimen (AHFCC-1SF and AHFCC-2SF) were first made according to a mixture spread of 160 mm, which provided insufficient mixture workability to enable the alignment of the fibers in the cementitious mixture. Therefore, to optimize the mixtures workability of the cementitious composites for an efficient alignment process, different mixtures’ workability were then investigated, namely, mixtures spread with diameters of 160 mm, 200 mm, 240 mm, and 280 mm, respectively. For that purpose, the specimens were all made in cylindrical molds (with 100 mm as diameter and 50 mm as length) with a content of 0.81 g of hybrid fibers corresponding to a volume fraction (*V*_f_) of 0.12%. The intended alignment direction of the hybrid fibers was indicated on each mold, which was then placed in the magnetic chamber, parallel to the magnetic field where an electromagnetic intensity of 30 V corresponding to 0.012 T was applied within a period of 70 s. Finally, in order to check the effectiveness of the alignment process, both specimen types (AHFCC-1SF and AHFCC-2SF) were orthogonally split to the alignment direction of the hybrid fibers after 24 h using a splitting tensile test. Then, the average amount of aligned hybrid fibers obtained across the induced crack-section into each specimen was determined.

**Figure 4:**
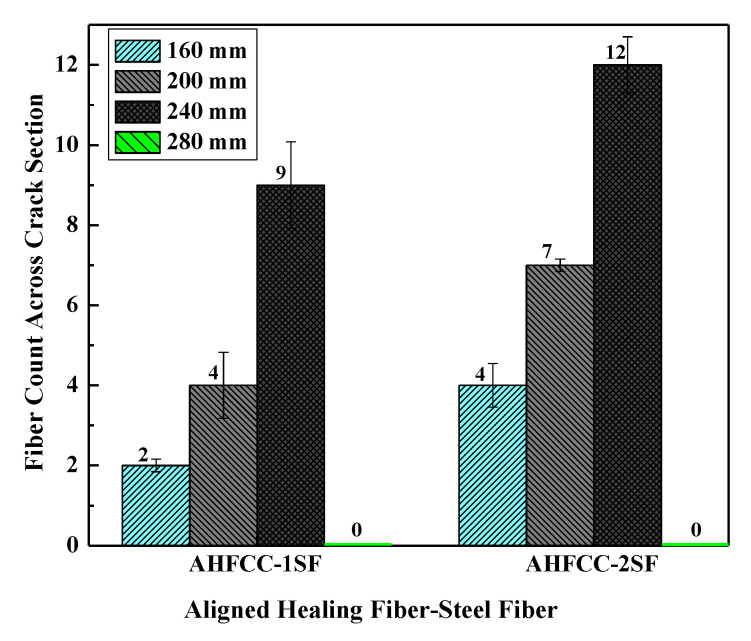
Optimal requirements analysis for the automatic alignment process.

Thus, according to the distribution across the crack sections, a random distribution of the hybrid fibers into the crack matrix with single fibers and agglomerated fibers was observed. The average amounts of aligned hybrid fibers obtained across the crack section in each specimen type are shown in Figure 4. It can be noticed that aligned hybrid fibers have been obtained with a higher value of the mixture spread of 240 mm for both specimen types (50% for AHFCC-2SF and 37% for AHFCC-1SF), which indicates that the mixture workability influences the alignment process. However, according to the combined amount of steel fiber with the healing fibers in the mixture spread of 240 mm, AHFCC-2SF achieved the higher ratio of aligned hybrid fibers. Then, it can also be noticed in Figure 4 that unaligned hybrid fibers have been obtained in the mixture spread of 280 mm, due to its very low mixture viscosity, which led all the hybrid fibers in the mixture of both specimen types to rise to the surface. This is the reason why the column result of the mixture spread 280 mm was not observed in Figure 4. This point was emphasized in a prior study, saying that the viscosity of the mixture should not be too low, in order to prevent the fibers from sinking [13]. It can be deduced that the specimen AHFCC-2SF containing healing fiber with double steel fibers, made with a mixture spread of 240 mm, allowed the most efficient alignment process using a magnetic intensity of 0.012 T within an applied period of 70 s. Therefore, these criteria were adopted as the optimal requirements for the alignment process of the healing fibers during the manufacturing of the main specimens for the self-healing test in the following sections of this research.

### 3.2. Healing Fiber Amount across a Crack Section in AHFCC-2SF and RHFCC

To analyze how the alignment of the healing fibers can improve the self-healing method in cementitious composites, an assumption stating the amount of the healing fibers that can be obtained across a crack section was initiated as the key point to improve the possible crack width that can be healed. Investigations were undertaken to determine the amount of healing fiber that can be immersed through a crack cross-section based on the healing fibers’ orientation in the cementitious matrix. The only purpose of using the hybrid fibers in this study was to enable an automatic alignment of the healing fibers into the specimen matrix, so hybrid fiber was the container type used in the specimens AHFCC-2SF to be able to effectively align the healing fibers into the specimen matrix based on the prior investigations on the alignment process in such material. Then, only healing fibers instead of hybrid fibers were used in the specimen RHFCC to obtain randomly distributed fibers in the cementitious composite. Thus, the obtained average amounts of fibers across a crack section in both specimen types (AHFCC-2SF and RHFCC) were used later for the specimen manufacturing for the self-healing process. The specimen’s mixtures containing aligned hybrid fibers were entirely made according to the optimal requirements for an effective alignment process as previously determined. The requirement was to combine each healing fiber with double steel fibers (AHFCC-2SF) made with a mixture spread of 240 mm. The specimens containing randomly distributed healing fibers (RHFCC) were also made with the same mixture spread of 240 mm. Both specimen types were made in cylindrical molds (100 mm as diameter and 50 mm as length) with a content of 0.81 g of fibers per specimen, corresponding to a volume fraction (*V*_f_) of 0.12%. Knowing the alignment direction of the hybrid fibers in AHFCC-2SF, the specimens were orthogonally split to the alignment direction of the fibers, while the specimens RHFCC were split in a random direction. Finally, the amount of fibers across the induced crack section in each specimen type was determined from averages.

Thus, according to the test results, the AHFCC-2SF specimens obtained an average of 0.43 g (12 fibers) of hybrid fiber in the cross-section of the induced crack, which corresponded to 50% of the total fibers per specimen. Then, in the case of the RHFCC specimens, an average of 0.25 g (7 fibers) of healing fiber across the crack section was obtained, corresponding to 29% of the total fibers per specimen. It can be noticed that the AHFCC-2SF specimen obtained the most significant results as compared with the RHFCC specimen. In the same manner, it was confirmed that the container’s content ratio for the self-healing in a material can have a very significant effect on the healing efficiency by increasing the available volume of healing agent needed to heal the cracks [10]. The more of the encapsulated healing agent there is, the more the healing efficiency will be increased by filling the crack interface as much as possible. It can be seen through our study that aligning healing fibers in a cementitious matrix can allow more containers across a crack section, which can also contribute to increasing the available volume of healing agent needed to heal the crack. This was confirmed through a study showing that the main advantage of using tubular fibers is the possibility of multiple healing due to their length and their greater encapsulation capacity [10,11]. Such abilities make it possible to heal higher crack widths. Additionally, aligning tubular fibers in a composite matrix also intervenes as a significant factor influencing the self-healing process by increasing the possibility of several fibers being reached by cracks, which also contributes to healing larger crack widths.

### 3.3. Self-Healing Activation Process

The self-healing process proceeded by first manufacturing new specimen groups which were specimens containing aligned healing fibers in cementitious composites (AHFCC) and specimens containing randomly distributed healing fibers in cementitious composites (RHFCC). The mixtures of both specimen types were made according to a mixture spread of 240 mm, which was the previously requested optimal condition for the effective alignment of the healing fibers using a magnetic field. The healing fibers, without having been combined with steel fibers, were this time manually aligned in the AHFCC specimens, precisely in the cross-section of the intended applied load axis, following exactly their orientation angles and distributions, as obtained in the specimens’ crack cross-section in the prior test. In the RHFCC specimens, the healing fibers were also integrated into the cross-section of the intended applied load axis, following exactly their random distributions as obtained in the specimens’ crack cross-section in the prior test. The healing fiber content ratios used in each specimen type were the same as the ratios previously obtained across the crack section, which were, respectively, 0.43 g (*V*_f_ = 0.06%) for AHFCC and 0.25 g (*V*_f_ = 0.04%) for RHFCC.

**Figure 5:**
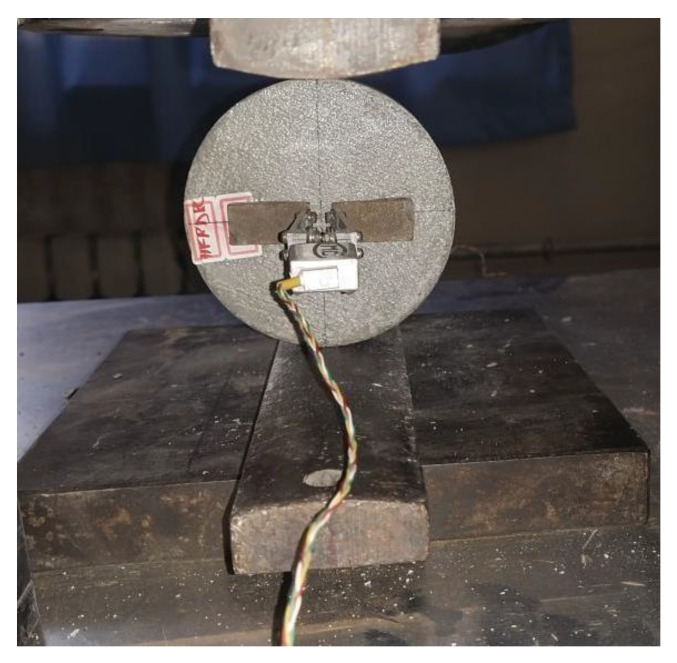
Splitting tensile test.

After the manufacturing and maturation period of the specimens, the self-healing activation process was conducted by applying a uniaxial load on the specimens, orthogonal to the direction of the healing fibers in both specimens’ matrixes, using a compressive machine test as shown in Figure 5. Two steel cushions between each flat loading platform were used to diametrically compress the specimen in the planned crack direction until failure. Two clip gauges with 20 mm as maximum width were hanging on each specimen at its two main faces using two steel shims fixed at 10 mm from each other at the disc center. The spacing of the clip gauges and the loading stress were recorded at the same time during the test and the clip gauges data were converted to the real width value using a calibration equation. Then, the averages of the crack width obtained from the clip gauges at both faces were automatically determined to be used as a signal to unload the specimens when the intended crack width was reached according to a range of [200–300 μm]. Therefore, during the inducing of the crack into the specimen matrix, the healing fibers were broken and the healing agent contained in the reached fibers was released into the crack. According to the prior tests, the setting time of the healing agent requested to activate the self-healing process within 10 days maximum. Therefore, to ensure a good flow of the healing agent into the crack interface for an effective healing, the self-healing process was activated 7 days after manufacturing of the specimens.

## 4. Healing Efficiency of Aligned Tubular Fibers Full of Healing Agent

### 4.1. Specimen Recovery According to the Splitting Tensile Strength

**Figure 6:**
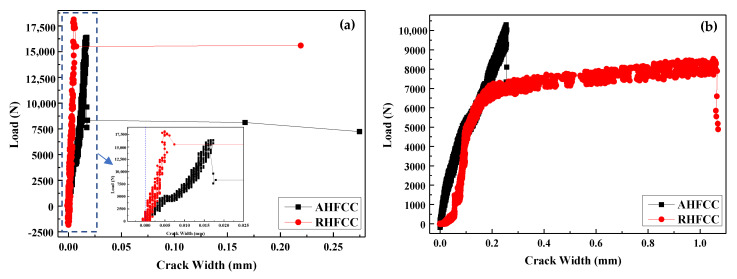
Splitting tensile test analysis: (**a**) load–crack width curve before healing, (**b**) load–crack width curve after healing.

**Figure 7:**
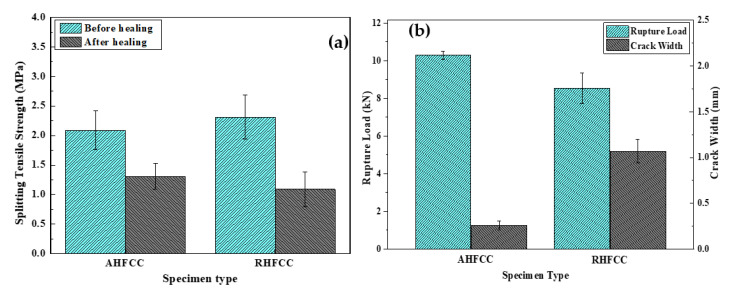
Healing capability evaluation: (**a**) splitting tensile strength before and after healing, (**b**) load–crack width relationship after healing.

Figure 6a shows the load–crack width curves of both specimen types during the splitting tensile test before healing. It can be observed that in each case, the load was continuously applied throughout the test, increasing until reaching the maximum load from where the crack occurred. Then, the applied load in each case decreased until becoming approximately constant, while the crack width kept increasing until unloading of the specimen. Thus, AHFCC obtained a maximum crack width of 270 μm for a maximum load of 16.39 kN, which corresponds to 2.09 MPa as splitting tensile strength, while RHFCC obtained a maximum crack width of 220 μm for a maximum load of 18.13 kN, which corresponds to 2.31 MPa as splitting tensile strength, as shown in Table 2. Compared to the AHFCC specimen stiffness, an increase of about 10% was observed in the RHFCC specimen stiffness, which was due to the higher amount of healing fibers contained in the AHFCC specimen, all aligned orthogonally to the crack direction. This may have favored the earlier initiation of cracks in AHFCC. Figure 6b and Figure 7b show the load–crack width curve after healing and a relationship analysis between the rupture load and crack width after healing. It can be observed that AHFCC developed a rupture for a maximum load of 10.29 kN, corresponding to 1.31 MPa as splitting tensile strength, which induced a crack width of 260 μm, while RHFCC developed a rupture for a maximum load of 8.54 kN, corresponding to 1.09 MPa as splitting tensile strength, which induced a crack width of 1070 μm, as shown in Table 2. According to the rupture loads and the width of the induced cracks after healing, it can be observed that AHFCC obtained the higher peak load, with the lower crack width. Figure 7a shows a comparative analysis of the specimen’s splitting tensile strength before and after healing; it can be observed that after healing, AHFCC recovered about 63% of its splitting tensile strength before healing, while RHFCC recovered only 47% of its splitting tensile strength before healing. The prior observations show that AHFCC effectively recovered much of its stiffness, due to the higher available volume of the healing agent obtained from the healing fiber’s amount across the crack section. This has been possible by aligning the healing fibers in the cementitious composite, parallel to the direction of the splitting tensile stress.

### 4.2. Specimen Recovery According to the Permeability

**Figure 8:**
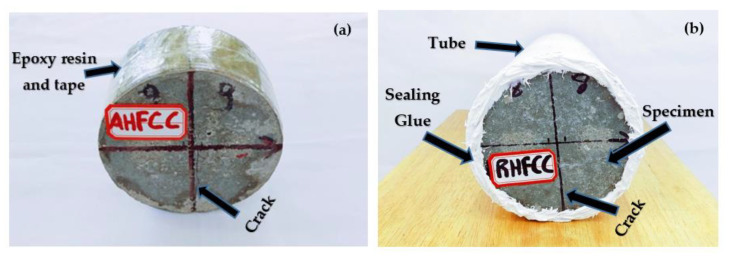
Specimen preparation for the permeability test: (**a**) a specimen’s lateral face sealing with epoxy resin and adhesive tape, (**b**) circumference’s sealing of the PVC tubes containing the specimen.

**Figure 9:**
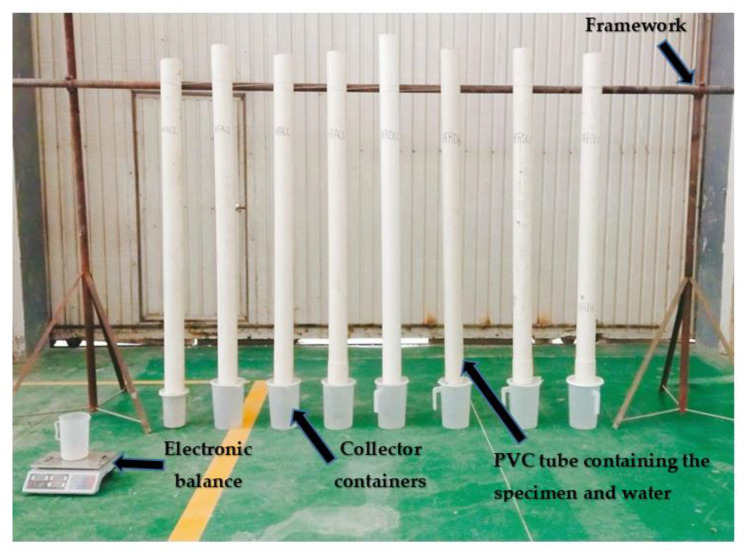
Framework for the permeability test.

After having induced a crack in the specimens for activation of the self-healing process, the specimens were kept at an ambient temperature for 7 days to allow the healing agent to harden enough before conducting the permeability test. Then, epoxy resin was applied on the lateral face of each specimen, as shown in Figure 8a, to fill the pores, including parts of the induced crack on the lateral side. After 7 days, the specimens were each wrapped with an adhesive tape before being inserted into PVC tubes with an inner diameter of 100 mm and 2000 mm as length. The circumference between the tubes and the specimens was sealed using glue, as shown in Figure 8b, to make sure that there was only water flowing from the crack at the specimens’ main faces. After preparation of the specimens, the permeability test was achieved by fixing the tubes containing the specimens on a framework, as shown in Figure 9, then the tubes were wholly filled with a total water volume of 1.178 × 10⁻^2^ m^3^. Containers were placed under each tube to collect the water flow from the cracks. The collected water flow was recorded every 40 min by measuring the weight of each container’s content, to determine the accurate corresponding volume of the collected water flow and then the container’s content was immediately returned into the tubes to keep constant the applied pressure on the specimens until the rate of the volume water flow became constant. Finally, according to the assumption of water flow through a system for being continuous and laminar, Darcy’s law was applied. Darcy’s coefficient of permeability was determined using Equation (1) [14]:

K = QL/AΔP(1)

where Q is the water flow rate through the specimen (m^3^/s), K is Darcy’s coefficient of permeability (m/s), A is the cross-section of the sample (m^2^), ΔP is the difference in pressure due to the water height (Pa), and L length of the sample (m). In this manner, the obtained results allowed the following analysis.

**Figure 10:**
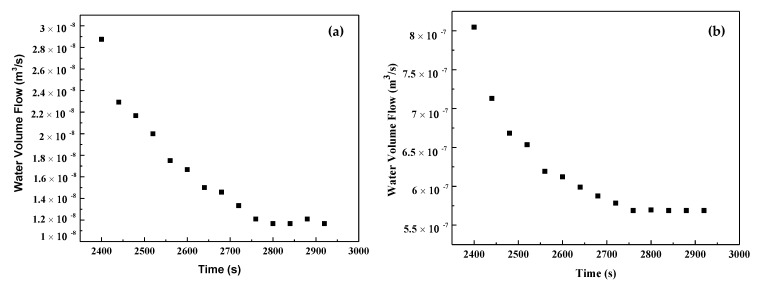
Water volume flow rate over time, (**a**) AHFCC and (**b**) RHFCC.

**Figure 11:**
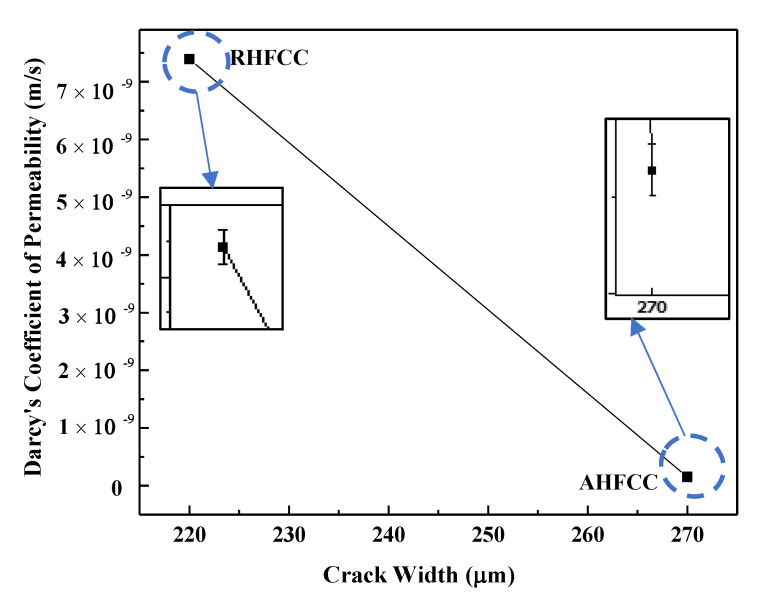
Darcy’s coefficient of permeability K according to specimen type.

Figure 10a,b shows the volume flow rate of AHFCC and RHFCC specimens over time. It can be observed that, in both cases, the volume flow rate decreased over time until reaching a constant value. In addition, the volume flow rate through AHFCC was lower than in the case of RHFCC. Darcy’s coefficient permeability K was determined through the Darcy’s law from the constant phase of each curve. Knowing that during the splitting tensile test before healing, the obtained crack widths for each specimen type were 270 μm for AHFCC and 220 μm for RHFCC, as shown in Table 3, the obtained volume flow rate during the permeability test in AHFCC was expected to be higher than in RHFCC. In prior works, it was shown that in additional to factors such as the pores, the crack width has a predominant effect on the permeability of a material [15,16]. It was also indicated that being in a material matrix, the crack constitutes a preferential way for any infiltrated liquid in the matrix [17,18]. Figure 11 shows the coefficient permeability K of the specimens (AHFCC and RHFCC) according to the crack width—it was observed that RHFCC presented a permeability of 7.39 × 10⁻^9^ m/s, 98% higher than AHFCC, with a permeability of 1.53 × 10⁻^10^ m/s, as shown in Table 3. From the obtained results, it can be deduced that according to the permeability of both specimen types, AHFCC containing aligned healing fibers provided a better permeability property. This can be explained by the influence of the healing process, through which the aligned healing fibers obtained in the specimen crack cross-section of AHFCC have released enough healing agents into the crack interface, which was not the case for the RHFCC specimen.

## 5. Discussion

### 5.1. Influence of the Alignment of Hybrid Fiber on the Composite Mechanical Performance

The influence of the healing fibers on the specimens’ mechanical properties was analyzed through a compressive test. For that, mainly three types of specimens were made with different healing fiber content ratios, which were 0% healing fibers for the specimen control (RC), 0.81 g of hybrid fibers corresponding to a volume fraction (*V*_f_) of 0.05% for the specimen (24AHFCC-2SF), and 1.16 g of hybrid fibers corresponding to a volume fraction (*V*_f_) of 0.07% for the specimen (30AHFCC-2SF). These fiber ratios were specifically chosen based on the content ratio used for the self-healing test. It was mainly intended to determine the decreasing effect of the container content of 0.81 g used for the self-healing process, and then to compare it to the healing efficiency obtained with the same container ratio. Additionally, the volume fractions (*V*_f_) of 0% and 0.07% were used to analyze the influence of increasing the healing fiber ratio in a composite, to determine the maximum ratio that should not be exceeded, to minimize as much as possible the healing fiber’s effect on the composite mechanical properties, and to compare the healing fiber’s effect on the mechanical properties of a cementitious matrix without any fiber content. Cubic plastic molds 100 mm in size were used to make the specimens. According to the optimum required conditions for the effective alignment process previously determined, the hybrid fibers (healing fiber combined with double steel fibers) were aligned with double steel fibers in the cementitious mixtures (24AHFCC-2SF) and (30AHFCC-2SF). Finally, the specimens were tested after 28 days to determine their compressive strength and rupture load from averages.

**Figure 12:**
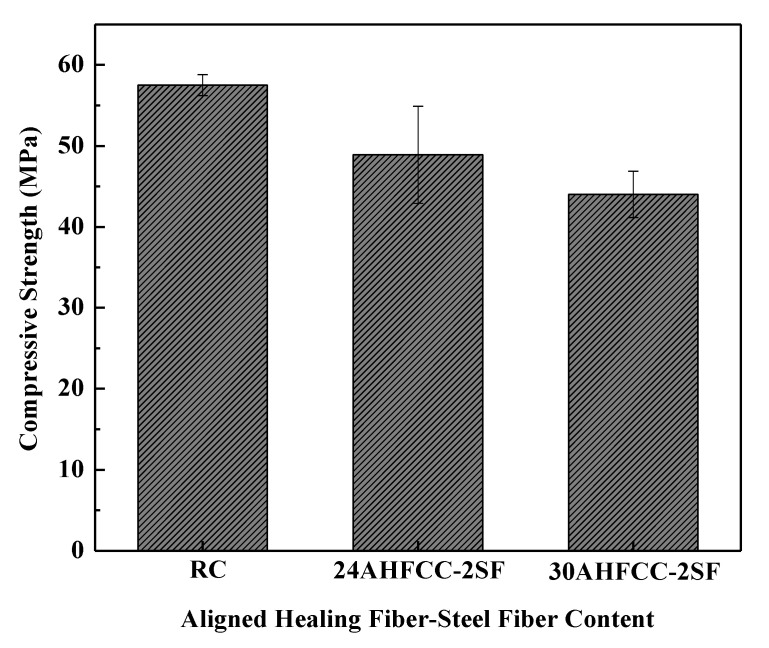
Healing fiber effect analysis on a cementitious composite.

Thus, Figure 12 shows the comparative analysis of the compressive strength of a cementitious matrix according to the healing fiber contents. It can be observed that the 24AHFCC-2SF (55.6 MPa) and 30AHFCC-2SF (44 MPa) specimens had lower compressive strengths compared to the control specimen, RC (57.5 MPa). However, regardless of the strength of the control specimen, RC, it can be observed that there was a decrease of only 3% of the compressive strength in the 24AHFCC-2SF specimen, while in the case of the 30AHFCC-2SF specimen we obtained a decrease of 24%. This indicates that the healing fibers start to significantly affect the material’s compressive strength from a fiber content ratio up to 0.81 g. This is consistent with prior works, which noticed, from research, a decrease in the compressive strength of the mortar matrix according to an increase of the container’s content ratio [10]. It has been indicated that the use of containers such as microcapsules or healing fibers can affect the mechanical properties of a material matrix. However, it was indicated that the container’s content ratio’s influence on the mechanical performance of a material can eventually be neglected compared to the relationship between the ratio of the reduced performance and the ratio of the healing efficiency [10].

### 5.2. Practical Use of the Self-Healing Method in the Construction Field

The principle of aligning tubular containers containing healing agents into a composite material for self-healing has, in fact, been the subject of prior investigations. However, this method still needs extensive investigation for an effective application in practical cases. This study mainly focused on an automatic method, allowing for the alignment of tubular containers containing a healing agent in a cementitious matrix for self-healing. Therefore, based on the results, the study implementation in practical use needs to be shown, providing points such as the method and the limitations.

In a real case, the self-healing method presented in this study suggests using mainly hybrid fibers in the cementitious mixture for the container for encapsulation, with a device that allows applying a sufficient magnetic field to automatically align the fibers according to the intended direction in the structure. For that, the tubular container needs to first be combined with steel fibers in order to allow both fibers to be dragged by the applied magnetic field. Additionally, suitable mixture workability should be determined to enable the alignment process and avoid the sinking of the fibers in the mixture, considering the intended water to cement ratio. A container content ratio that does not reach up to 0.12% should be used in the mixture in order to limit the decreasing effect on the structure’s mechanical performance. 

However, the indicated method also presents some limitations that could constitute obstacles to its effective realization for practical use. In fact, the preparation of the hybrid fiber by combining steel fibers with each container requires an automatic method to efficiently realize this step. In addition, using hybrid fibers as the container may introduce the problem of the fibers being effectively broken when a crack occurs in the structure, due to the high elastic strength of the steel fibers, so the healing process may not happen.

In order to remedy such problems, magnetic particles may be integrated during the container manufacturing to obtain a shell that is able to react to an applied magnetic field. Thus, after encapsulation of the healing agent in the container, they can be directly integrated into the mixture and dragged by the magnetic field due to the magnetic particles in the container shell, which will allow the container to easily be broken when a crack occurs.

## 6. Conclusions

This research presents an analysis of the efficiency of an autonomic self-healing process using healing fibers (PVDF) aligned by a magnetic field in a cementitious composite. The process of the alignment method was first investigated. Then, the healing capability was analyzed through a repetitive splitting tensile test and a permeability test according to the healing fibers’ orientation in the composite matrix, which was aligned healing fibers (AHFCC) and randomly distributed healing fibers (RHFCC). The effect of the healing fibers on the cementitious composite was analyzed through a compressive test according to different healing fibers’ content ratios. Finally, the healing capability has been compared with the effect of the healing fiber on the specimens’ mechanical properties. The prior results allow the following conclusions:

- The analysis of the optimum requirements for an efficient alignment process using a magnetic field showed an effective influence in using a hybrid fiber (combination of two steel fibers with each healing fiber) for the alignment process. Additionally, in regards to the quantities of the combined steel fiber, the mixture’s workability also intervenes as a key element for an effective automatic alignment process using a magnetic field. The mixture workability should be lower than the magnetic field, to allow an effective alignment of the healing fibers. However, a mixture with a viscosity too low will, on the contrary, lead the fibers to sink into the mixture.
- The healing fibers’ orientations showed an effective influence on the healing capability, with the aligned healing fibers providing a more effective recovery than with the randomly distributed healing fibers, according to a repetitive splitting tensile test before and after healing, as well as permeability properties. This is due to the amount of the containers that penetrate the crack cross-section through the alignment of the healing fibers in the composite.
- Additionally, the presence of healing fibers in the matrix of the cementitious composite showed a decreasing effect on the material’s mechanical properties. However, according to the research results and based on previous literature, it has been deduced that the healing fibers’ effect on the mechanical properties can be neglected compared to the healing capability obtained by aligning the healing fibers parallel to the direction of the tensile stress in the cementitious composite matrix.
- In the case of this research, the self-healing activation process requested a maximum time of up to 10 days, due to the setting time of the healing agent to ensure an effective healing process. This setting time may differ from other cases according to the circumstance.

Despite these research results based on the study methodology, a fundamental factor that can improve the self-healing performance, which is the conservation time of the healing agent into a composite matrix, needs to be investigated further in future research.

## Figures and Tables

**Table 1:** Mixture design for the self-healing process.

Specimen Type	W/C	Cement (kg/m^3^)	Sand (kg/m^3^)	Water (kg/m^3^)	Super Plasticizer (kg/m^3^)	Spread (mm)	Distribution of Healing Fiber
AHFCC	0.36	651	1302	235	1.34	240	Aligned
RHFCC	0.36	651	1302	235	1.34	240	Random

**Table 2:** Results of the splitting tensile test before and after healing.

Specimen Type		Average Crack Width (μm)	Healing Fiber Content (%)	Steel Fiber Content (%)	Splitting Tensile Strength (MPa)
Before healing	AHFCC	270	0.06%	0.08%	2.09
	RHFCC	220	0.04%	0.08%	2.31
After healing	AHFCC	260	0.06%	0.08%	1.31
	RHFCC	1070	0.04%	0.08%	1.09

**Table 3:** Permeability test results.

Specimen Type	Average Crack Width (μm) before Healing	Average Water Flow Q at Constant Phase (m^3^/s)	Darcy’s Coefficient of Permeability K (m/s)
AHFCC	270	1.183 × 10⁻^8^	1.53 × 10⁻^10^
RHFCC	220	5.689 × 10⁻^7^	7.39 × 10⁻^9^

## Data Availability

Available on request from the corresponding author.
